# Patient-specific modeling of right coronary circulation vulnerability post-liver transplant in Alagille’s syndrome

**DOI:** 10.1371/journal.pone.0205829

**Published:** 2018-11-08

**Authors:** Miguel Silva Vieira, Christopher J. Arthurs, Tarique Hussain, Reza Razavi, Carlos Alberto Figueroa

**Affiliations:** 1 Division of Imaging Sciences & Biomedical Engineering, King's College London, London, United Kingdom; 2 Department of Pediatrics, University of Texas Southwestern Medical Center at Dallas, United States of America; 3 Pediatric Cardiology Department, Evelina Children’s Hospital London, Guy’s and St. Thomas’ NHS Foundation Trust, London, United Kingdom; 4 Departments of Surgery and Biomedical Engineering, University of Michigan, Michigan, United States of America; Indiana University, UNITED STATES

## Abstract

**Objectives:**

Cardiac output (CO) response to dobutamine can identify Alagille's syndrome (ALGS) patients at higher risk of cardiovascular complications during liver transplantation. We propose a novel patient-specific computational methodology to estimate the coronary autoregulatory responses during different hemodynamic conditions, including those experienced in a post-reperfusion syndrome (PRS), to aid cardiac risk-assessment.

**Material and methods:**

Data (pressure, flow, strain and ventricular volumes) from a 6-year-old ALGS patient undergoing catheter/dobutamine stress MRI (DSMRI) were used to parameterize a closed-loop coupled-multidomain (3D-0D) approach consisting of image-derived vascular models of pulmonary and systemic circulations and a series of 0D-lumped parameter networks (LPN) of the heart chambers and the distal arterial and venous circulations. A coronary microcirculation control model (CMCM) was designed to adjust the coronary resistance to match coronary blood flow (and thus oxygen delivery) with MVO2 requirements during Rest, Stress and a virtual PRS condition.

**Results:**

In all three simulated conditions, diastolic dominated right coronary artery (RCA) flow was observed, due to high right ventricle (RV) afterload. Despite a measured 45% increase in CO, impaired coronary flow reserve (CFR) (~1.4) at Stress was estimated by the CMCM. During modeled PRS, a marked vasodilatory response was insufficient to match RV myocardial oxygen requirements. Such exhaustion of the RCA autoregulatory response was not anticipated by the DSMRI study.

**Conclusion:**

Impaired CFR undetected by DSMRI resulted in predicted myocardial ischemia in a computational model of PRS. This computational framework may identify ALGS patients at higher risk of complications during liver transplantation due to impaired coronary microvascular responses.

## Introduction

Alagille’s syndrome (ALGS) is a rare autosomal dominant multi-systemic vasculopathy, with variable penetrance and expression, and an estimated incidence of 1:30,000 up to 1:50,000 per liver birth [1,2]. Several mutations have been reported in genes involved in the Notch signaling pathway that regulate differentiation of cell migration during fetal vascular development [3]. These are thought to be the cause of this polymalformative disorder affecting the liver (paucity of interlobular bile ducts resulting in neonatal cholestasis), heart (peripheral pulmonary stenosis, PPS), eyes (posterior embryotoxon), and skeleton (butterfly-like vertebral arch defects), and associated with a characteristic dysmorphic facies. Currently, genetic testing is available and enables non-invasive confirmation since the phenotypic expression of the disease is highly variable [4]. ALGS typically presents in the first 3 to 6 months of life with cholestasis, coursing in half of the cases with debilitating pruritus, disfiguring xanthomas and failure to thrive due to fat malabsorption [4]. Despite aggressive medical care (e.g. tailored diet with carbohydrate, medium-chain triglycerides and individual fat-soluble vitamins supplementation, bile flow stimulants, bile acid–binding resins and antihistamines), in 20–30% of children liver transplantation (LT) is the only option for end-stage liver failure and the debilitating cholestasis symptoms [5].

In general, LT is associated with hemodynamic instability notably during the reperfusion of the allograft due to the sudden release of vasoactive mediators into the systemic circulation [6]. These events, first defined by Aggarwal S et al. [6] in 1987 (post-reperfusion syndrome, PRS) include, on one hand, a decreased in mean arterial pressure (MAP) and systemic vascular resistance (SVR), and on the other hand, an increase in pulmonary arterial pressure (PAP), pulmonary artery wedge pressure (PAWP), and central venous pressure (CVP), in addition to cardiac arrhythmias.

LT in ALGS has been associated with higher risk of complications (early/late mortality and graft failure), particularly during the allograft reperfusion. This increased risk has been ascribed to preexisting cardiopulmonary disease [7]. It has been postulated that PPS, which occurs in over 2/3 of patients, leads to progressive right ventricular (RV) remodeling (compensatory hypertrophy initially and eventually dilation with systolic function impairment). This on one hand, may result in acute right heart failure (RHF) and eventually cardiogenic shock during the critical period of reperfusion and, on the other hand, may prevent a sustained increase in CO normally required to perfuse the transplanted liver [7–11].

### Pre-transplant cardiovascular risk assessment through dobutamine stress imaging

A thorough pre-operative cardiac assessment is currently recommended to identify ALGS patients who are likely to be at higher risk because of their cardiovascular disease [12]. Razavi RS et al. [13] have proposed a dynamic method to mimic the hemodynamic conditions of reperfusion after LT. Using dobutamine as an inotropic vasodilator, they have shown that the patients’ hemodynamic response could predict their ability to increase the CO in the immediate post-transplant period to meet the demands of systemic hypotension that occurs with liver reperfusion [12,13]. Although not consensual, it has been proposed that a 40% increase in CO is the required hemodynamic response to the ensuing generalized systemic vasodilation in order to allow a successful liver transplant [12].

The standard protocol uses a two-stage dobutamine infusion (10 μg/kg/min and 20 μg/kg/min). This staged approach allows to assess the response to increasing doses and therefore prevent complications. Razavi RS et al. have described that a significant decrease in the systemic vascular resistance (close to that seen during liver reperfusion) was only observed with the higher dose of 20 μg/kg/min and thus this has been established as the target dose for a diagnostic test.

### Pressure-flow autoregulation and coronary flow reserve in ALGS

Under resting physiologic conditions, coronary blood flow (CBF) occurs predominantly during diastole, when intra-myocardial tissue pressure, particularly in the endocardium, falls below the aortic root pressure [14,15].

Notably, normalized resting CBF to the RV, which is significantly lower than that to the left ventricle (LV) due to its lower myocardial oxygen consumption (MVO_2_), is not significantly affected by its contraction. Due to lower RV systolic extravascular compressive forces, flow in the right coronary artery (RCA) occurs throughout the cardiac cycle [15,16]. In contrast, flow in the left coronary artery (LCA) flow is predominantly diastolic.

A complex network of integrative pathways not fully understood (metabolic, myogenic and endothelial-dependent), modulates the coronary microcirculation vasomotor tone, exerting a delicate control of the myocardial perfusion so that there is a close match between oxygen delivery and changes in MVO_2_ [17,18]. Myocardial perfusion is directly proportional to the pressure gradient across the coronary circulation and inversely proportional to coronary resistance. Given the heart’s limited anaerobic capacity and oxygen extraction reserve, notably reduced in RV pressure overload, often seen in ALGS patients, coronary vasodilation in response to inadequate myocardial oxygen delivery is critical to mitigate ischemic injury [15,19]. Despite a high degree of coronary microcirculation autoregulation, there is a threshold below which a further fall in the aortic perfusion pressure (e.g. during PRS) cannot be compensated by an additional decrease in coronary resistance. Moreover, the coronary vasodilator reserve appears to be lower in the RCA territory [20].

During physiological autoregulation, CBF changes in response to a decrease in MAP are the result of both a reduced perfusion pressure and a reduced MVO_2,_ consequence of a lower LV afterload. This strong coupling between CBF and MVO_2_ makes it difficult to study in-vivo complex coronary pressure-flow autoregulation mechanisms and isolate their individual contributions [15].

ALGS patients classically present some form of pulmonary vasculopathy (over 2/3 have PPS) [1,2]. As in other pulmonary vasculopathies, structural remodeling (stiffening) of the pulmonary arteries walls results in increased RV afterload and compensatory hypertrophy leading to a restrictive physiology [21]. Although to our knowledge no data is available in ALGS children, we hypothesize that with increasing RV wall tension and consequently RVMVO_2_, there is a progressive shift of RCA flow to diastole, rendering it more LCA-like. Van Wolferen SA et al. [22] have demonstrated in an MRI study that adults with pulmonary hypertension have systolic RCA flow impediment proportional to RV pressure and mass. This could predispose to RV ischemia and contribute to RV failure seen in some patients. In ALGS children with near-systemic RV pressures, a similar scenario may occur, with compression of the intramyocardial microvasculature reducing the perfusion gradient between the aorta and RCA bed and flow impairment occurring potentially even before the vasodilator reserve is exhausted [22–24]. This may increase the risk of RV ischemia and RHF during the dramatic cardiovascular and metabolic derangements of liver reperfusion, when the coronary perfusion pressure is further decreased [25].

### Rationale for the present study

We submit that the dobutamine stress test does not account for all key hemodynamic events during liver transplantation: while the test might reproduce the increase in heart rate and myocardial metabolic demand, it fails to account for the systemic vasodilation and corresponding coronary perfusion pressure drop during transplantation [26]. Furthermore, the general guideline of 40% increase in CO during dobutamine stress MRI (DSMRI) might not guarantee adequate RCA perfusion during post-reperfusion syndrome (PRS).

In this study, we propose to use computational modeling to shed some light into RCA hemodynamics during PRS, in combination with image-based and catheterization data on two subject-specific hemodynamic stages in an ALGS subject. Towards that, we study three different conditions:

1. *Rest* condition, in which a computational model of RV, LV, RCA, LCA, aorta, and pulmonary arteries is calibrated to reproduce baseline hemodynamic data for the patient.
2. *Stress* condition, in which the computational model above is adjusted to reproduce hemodynamic conditions during DSMRI for the same patient.
3. *PRS* condition, entirely computational, in which the Stress condition is further modified to account for the systemic pressure drop during PRS.

This framework can be used to study coronary flow reserve (CFR) and assess possible shortcoming of the DSMRI test.

## Material and methods

Ethical approval was obtained from St. Thomas' Hospital Research Ethics Committee/South East London Research Ethics Committee (10/H0802/65).

### 1. Catheter and magnetic resonance imaging study

Data was collected from a 6-year-old ALGS patient undergoing a hybrid X-ray catheter/DSMRI study as described previously [27]. Combined acquisition of functional (flow and volumes) at rest and peak dobutamine stress (20 μg/kg/minute) and three-dimensional (3D) morphologic MRI data (dual-phase), with concomitant central pressure monitoring, was performed and used to parameterize the computational simulations. Images were acquired using a 1.5T MR-scanner (Achieva, Philips, Best, Netherlands) and a Philips BV Pulsera cardiac X-Ray unit under general anesthesia, which was maintained constant throughout the procedure. Details of the imaging parameters are provided in Table A in S1 File.

### 2. Patient-specific computational modeling simulation of hemodynamics

A novel modeling framework that integrates image data and detailed invasive/non-invasive measurements was used to obtain a faithful computational representation of the patient’s physiology. A brief description of key aspects of the model is provided in the following sections (further details are available in the Technical Note in S1 File).

#### 2.1. Fluid-solid interaction models of the aorta and pulmonary arteries

**Fig 1:**
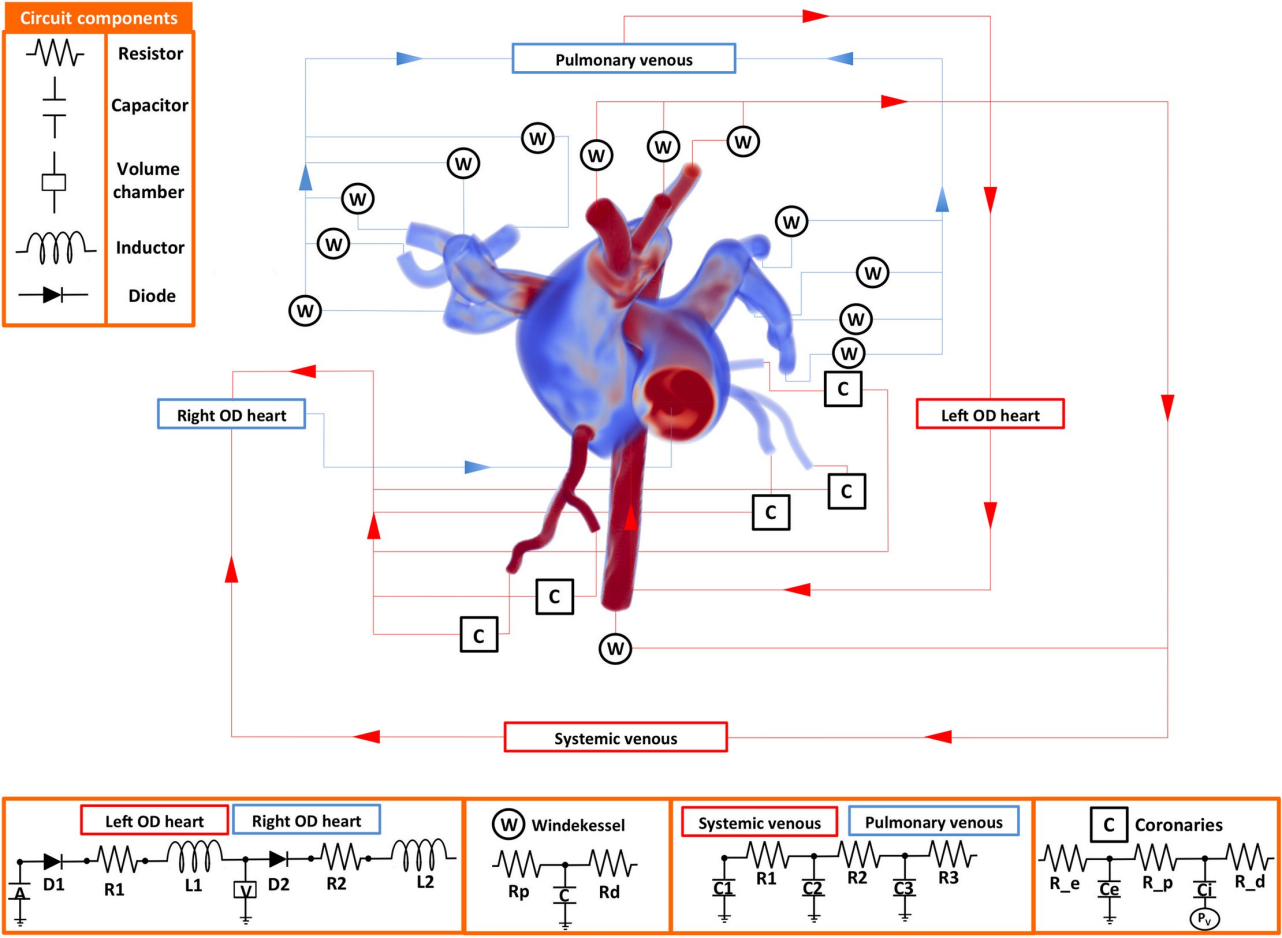
Patient-specific closed-loop model used to simulate pulsatile hemodynamics coupling lumped parameter networks (LPN, 0D) and image-based (3D) vascular models. The model includes: LV (0D), aorta, coronary arteries and major branches (3D), medium and small systemic arteries (0D), arterioles (0D), venules (0D), veins (0D), right atrium (0D), RV (0D), pulmonary arteries up to second generation branching (3D), small pulmonary arteries (0D), arterioles (0D), venules (0D), veins (0D) and left atrium (0D). P_v_ represents the node where the broadcasted intraventricular pressure was transmitted as described by Arthurs et al (2013) [18]. Details of the parameters used for each component can be found in the Technical Note in S1 File.

The CRIMSON custom software (CardiovasculaR Integrated Modeling and SimulatiON; http://www.crimson.software/) was used to segment the aorta and main branches, including the coronaries, and the central pulmonary arteries, using a semi-automatic segmentation method to produce an analytical representation of the vessels (Fig 1) from the diastolic phase of the 3D-Steady-state free precession (SSFP) sequence. Details regarding this segmentation approach are found elsewhere [28]. The 3D geometry was discretized into a volumetric finite element mesh consisting of 1,687,949 tetrahedral elements (characteristic dimension *h* = 0.8mm) and 325,463 nodes, with curvature-based refinement.

Blood was modeled as an incompressible Newtonian fluid (density *ρ* = 0.00106 g/mm^3^ and viscosity *μ* = 0.004g/mm·s). Blood vessels were modeled using the Coupled Momentum Method, whereby an incompressible linear elastic membrane with spatially-varying structural stiffness is monolithically-coupled to the fluid domain [29]. The linearized stiffness (*E*) of the aorta, its branches, and the central pulmonary arteries were prescribed according to the equation:

$$E=\frac{P_{syst}-P_{diast}}{D_{syst}-D_{diast}}\times D_{diast}\times h$$

where *P*_*syst*_ and *P*_*diast*_ represent the catheter-derived systolic and diastolic pressures, respectively, and *D*_*syst*_ and *D*_*diast*_ the 3D-SSFP-derived systolic and diastolic diameters at the level of the aortic and pulmonary artery roots, respectively. Wall thickness was prescribed to be 1 mm throughout. This produced a linearized stiffness of 2.26 x 10^5^ g/mm·s^2^ for the systemic and 1.30 x 10^5^ g/mm·s^2^ for the pulmonary arteries.

#### 2.2. Coupled multidomain model

Expanding on previous developments by our group [30], we have designed a closed-loop coupled-multidomain (3D-0D) model consisting of image-based portions of the pulmonary and systemic circulations (where image data was available), and LPN (0D) models of the heart chambers and the arterial and venous distal circulations (see Fig 1). Parameter values for the LPN models for each condition are given in Tables B-F of the Technical Note in S1 File.

#### 2.3. OD coronary microvascular control model (CMCM)

Metabolic control of the coronary microcirculation was achieved using a method develop by our group, which has been tested against invasive patient data [18]. The foundation of this LPN model relies on using CBF as a surrogate of myocardial oxygen delivery [18], and on dynamically adapting coronary microvascular resistance to eliminate any “myocardial hunger” (i.e. mismatch between oxygen demand and supply). The myocardial oxygen demand per heartbeat was computed from the cardiac workload, determined by the 0D heart model, after integrating the area of the ventricular pressure-volumes curves and the ventricular elastance function. Because the CMCM can reproduce patient coronary vasomotor responses to changes in cardiac workload, it has the ability to predict CBF adaptations in response to changes in the myocardial oxygen demand and coronary perfusion pressure gradient. This permits simulation of the coronary response to a generalized vasodilation state as seen during liver reperfusion and enables specific probing of the complex coronary microcirculatory responses.

The model assumed a fixed oxygen extraction of 40% and 80% for the RCA and LCA respectively at Rest and 100% for both coronary circulations at Stress [15,20].

#### 2.4. RV and LV elastance functions

RV and LV elastance functions were derived from ventricular cines and catheterization data, describing cyclic ventricular volume and intraventricular pressures, respectively, using methods described in Arthurs et al [18]. Ventricular volume curves, obtained by manually contouring the endocardial borders of all cardiac phases of the cardiac MRI short-axis cines, were matched with the corresponding R-wave in the invasive pressure recordings to obtain time-varying elastance functions (further details in the Technical Note in S1 File). Ventricular volumetric parameters for the Rest and Stress conditions used to define their corresponding elastance functions are listed in Table 1.

**Table 1:** RV and LV volumetric parameters for Rest and Stress conditions.

Condition	Heart Rate (bpm)	Mass index (g/m^2^)		EDV^a^ (ml/m^2^)		ESV^b^ (ml/m^2^)		EF^c^ (%)	
		LV	RV	LV	RV	LV	RV	LV	RV
**Rest**	73	54.2	59.8	81	90	35	44	60	51
**Stress**	106			80	95	23	37	71	61

^a^EDV, end-diastolic volume.

^b^ESV, end-systolic volume.

^c^EF, ejection fraction

#### 2.5. Additional specifications for the experimental conditions

At peak DSMRI, cardiac output increased by ~45% from baseline. No regional wall motion abnormalities were noted. Tissue stiffness was kept constant through all three experimental conditions. The PRS condition was defined by imposing a 31% drop in MAP relative to the Stress condition, following Hilmi et al. [10] definition of significant PRS.

All simulations were performed in our custom parallel blood flow solver CRIMSON using 128 processors on a SGI Altix UV, using a total simulation time of 150 hours per case.

## Results

**Fig 2:**
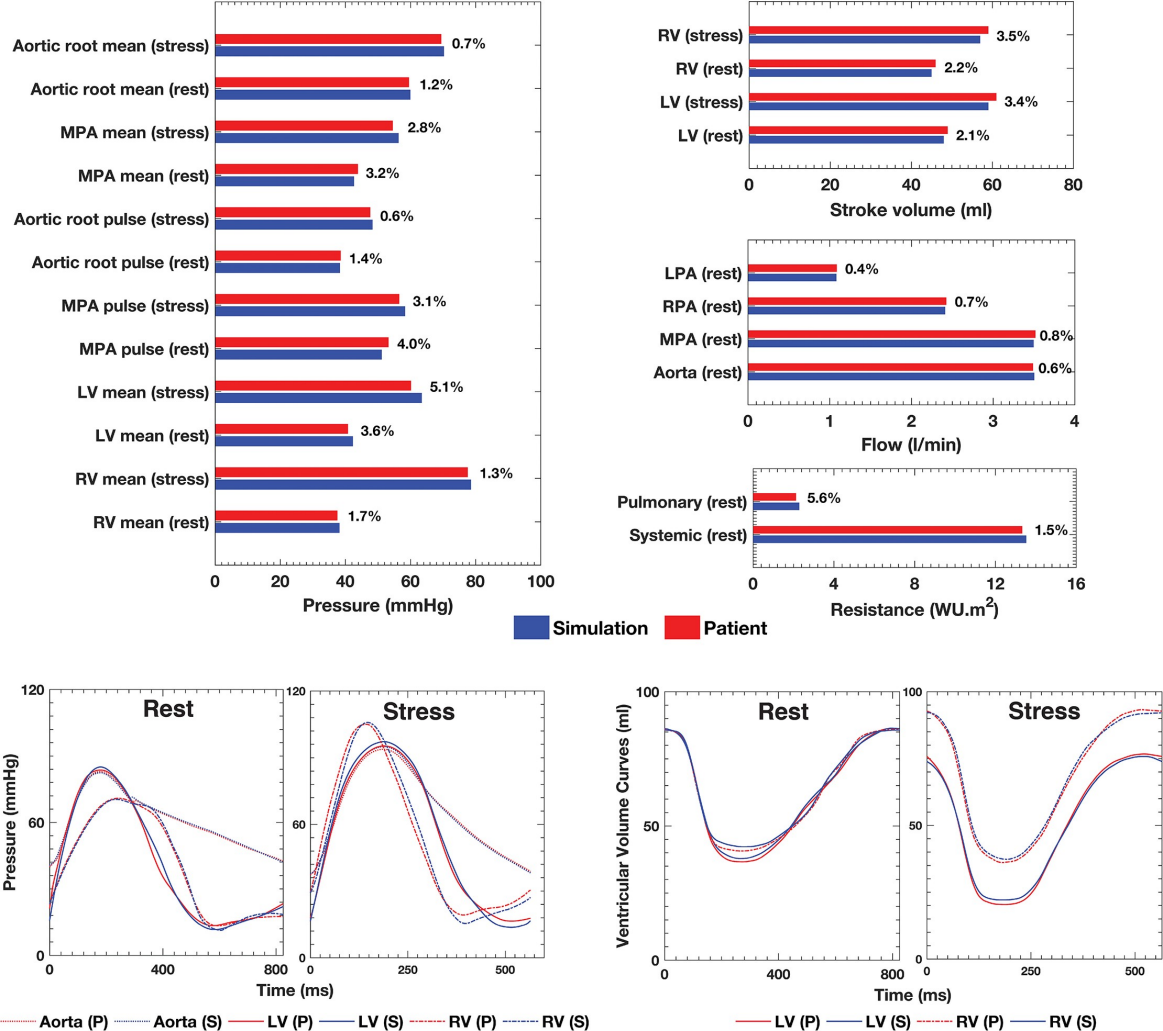
Relative error between patient and simulation data given as a percentage. Bar plots comparing the clinical pressure and flow at relevant anatomic landmarks, and ventricular stroke volume and systemic and pulmonary vascular resistance data at Rest and Stress, and corresponding simulation results. Selected pressures and ventricular volumes transient waveforms corresponding to the cycle-to-cycle equilibrium for the Rest and Stress Conditions are also show. P, patient clinical data. S, simulation results.

**Fig 3:**
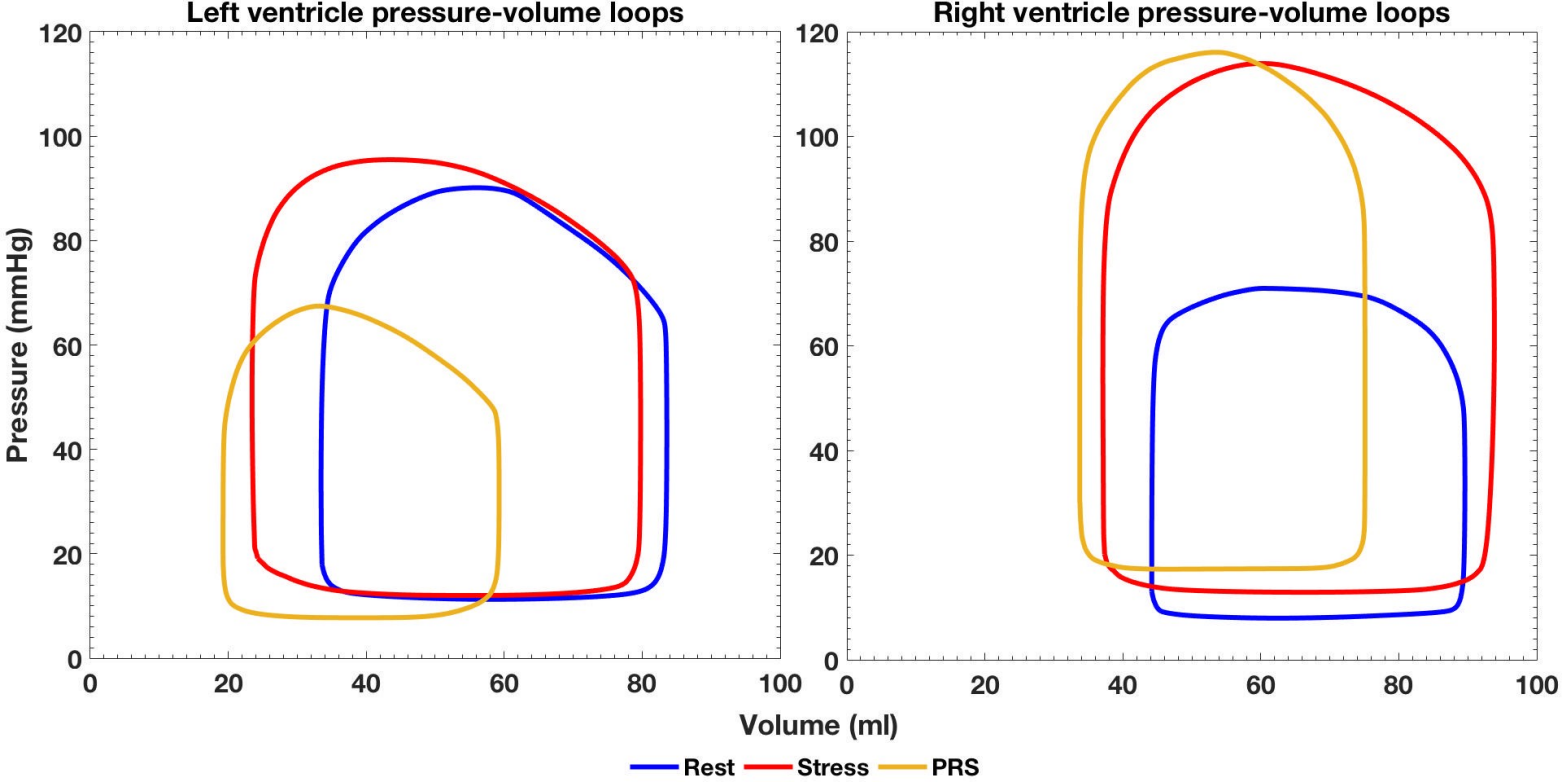
Left and right ventricle pressure-volume loops for the three simulated conditions.

Good agreement (< 6% discrepancy) between the clinical and simulated flow and pressure data was achieved (Fig 2). Tables 2 and 3 summarize relevant hemodynamic results from the simulation. Fig 3 depicts the LV/RV pressure-volume loops (PVL) computed for each condition. The PVL area was used to calculate the cardiac workload and MVO_2_ that are shown in Table 2. At Rest, LVMVO_2_ was just 6% higher than RVMVO_2_. At Stress, RVMVO_2_ was 12% higher than LVMVO_2_.

**Table 2:** Simulation results of cardiac workload and MVO_2_.

Condition	Cardiac workload (J)		MVO_2_ (ml/min)	
	LV	RV	LV	RV
**Rest**	4.6 x 10^−1^	3.5 x 10^−1^	5.42	5.10
**Stress**	5.2 x 10^−1^	7.2 x 10^−1^	6.97	7.93
**PRS**	2.7 x 10^−1^	4.9 x 10^−1^	4.39	8.91

**Table 3:** Simulation results of coronary hemodynamics including average CBF, coronary resistance and WSS.

Condition	Coronary blood flow (ml/min/g)		Diastolic/systolic flow ratio		Coronary Resistance (WU.m^2^)		Coronary WSS (dyn^.^cm^2^)	
	LCA	RCA	LCA	RCA	LCA	RCA	LCA	RCA
**Rest**	**66.2**	**67.6**	**1.8:1** (36.8:29.4)	**1.9:1** (35.6:32.0)	**1.07 x 10**^**2**^	**1.05 x10**^**2**^	**2.82**	**5.20**
**Stress**	**88.3**	**93.4**	**2.5:1** (53.0:35.3)	**2.7:1** (58.8:34.6)	**6.50 x10**^**1**^	**5.83 x 10**^**1**^	**5.33**	**24.18**
**PRS**	**51.5**	**44.6**	**0.7:1** (15.4:36.1)	**2.1:1** (23.4:21.2)	**2.36 x 10**^**1**^	**1.42 x 10**^**1**^	**3.34**	**15.61**

**Fig 4:**
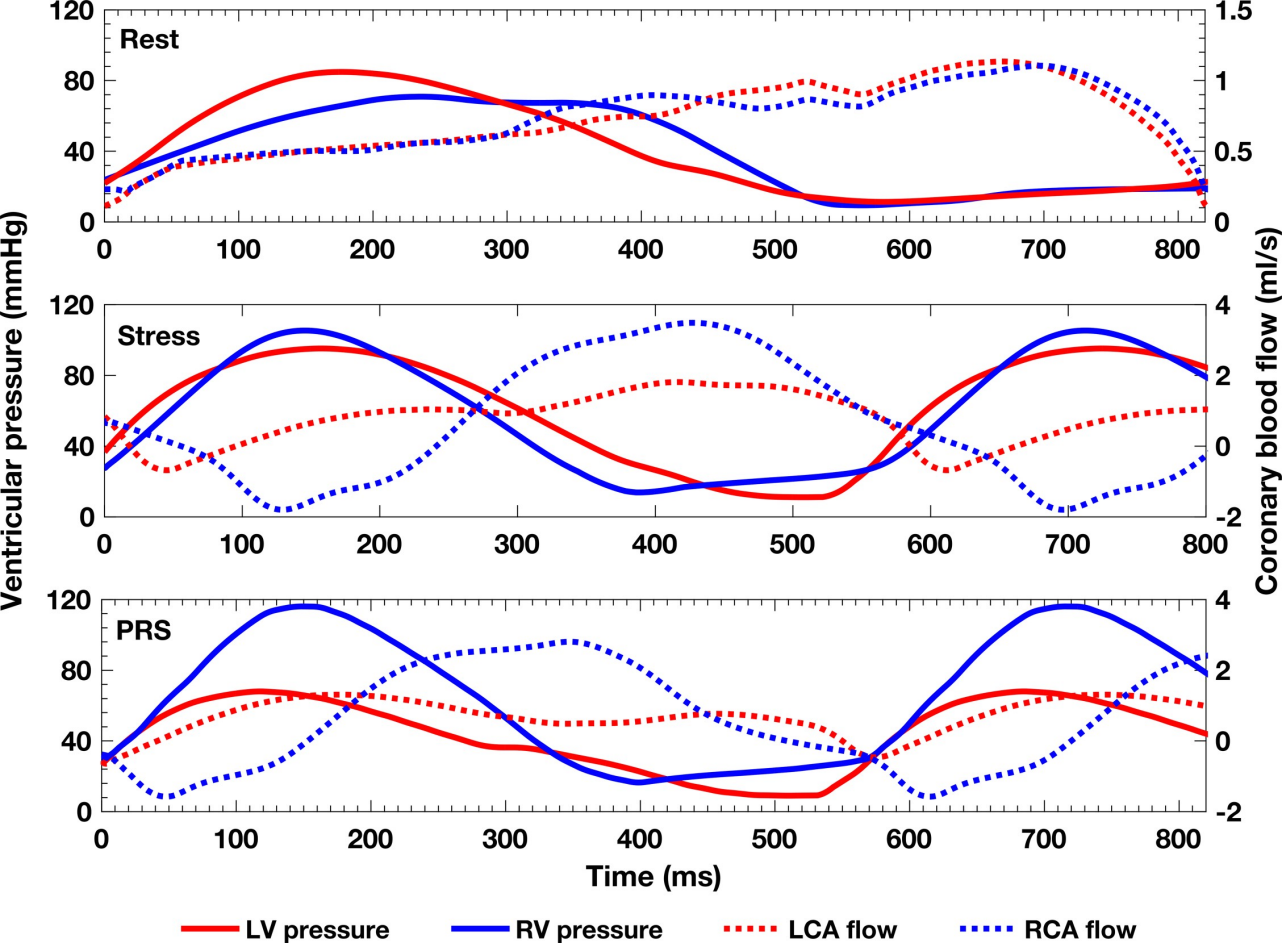
Left and right coronary flow waveforms and ventricular pressure for all three simulated conditions. Different y-axis scales were used in the Rest and Stress/PRS Conditions to best convey the waveforms pulsatility.

Table 3 shows several coronary indices computed from the simulation. The CMCM predicted a 1.4-fold increase in CBF with DSMRI (CFR ~ 1.4), followed by a 1.9-fold decrease during transplant PRS. Fig 4 illustrates the interplay between LV/RV pressures and coronary waveforms. In all three conditions, the coronary perfusion occurred mostly during diastole, except for LCA flow for the PRS condition. Diastolic RCA flow dominated, even at Rest. This RCA biphasic profile became more prominent during Stress and PRS conditions, with a dominant diastolic phase, marked systolic flow reversal, and higher peak diastolic-to-systolic flow ratios.

**Fig 5:**
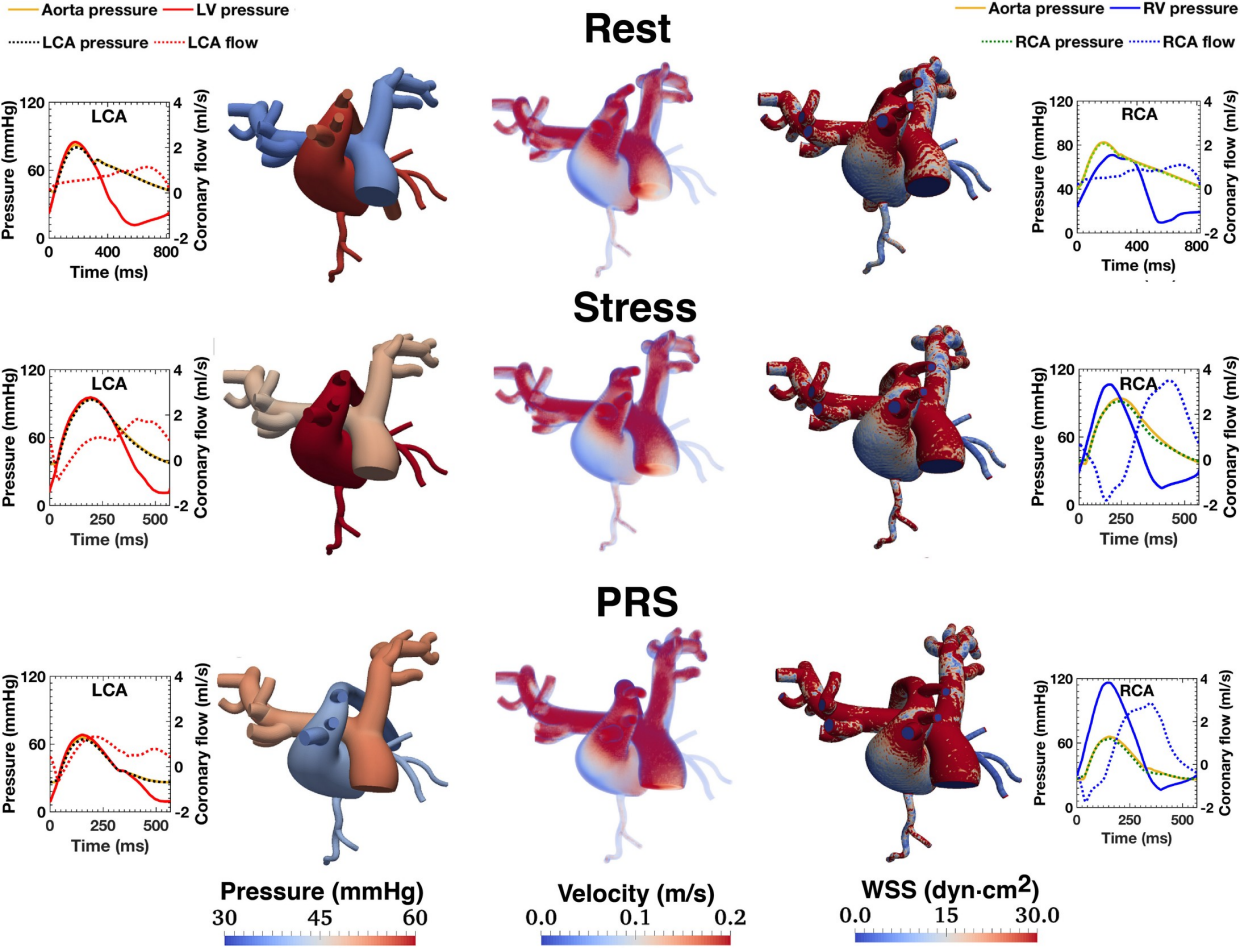
Time averaged maps of pressure, velocity and wall shear stress (WSS) (central panel) and selected flow and pressure waveforms (right and left panels). Notably, mean velocities and WSS in the RCA are higher than in the LCA. See also the Figure in S4 File with instantaneous 3D maps of pressure, velocity and wall shear stress (WSS) at peak systole and peak diastole.

Fig 5 presents the time-averaged 3D maps of pressure, velocity and wall shear stress (WSS) (central panel) and waveforms of aortic and ventricular pressure, as well as right and left coronary flows and pressures (left and right panels). In all three conditions, RCA mean velocity and WSS maps display higher values than in the LCA (see also the Movie in S2 File).

**Fig 6:**
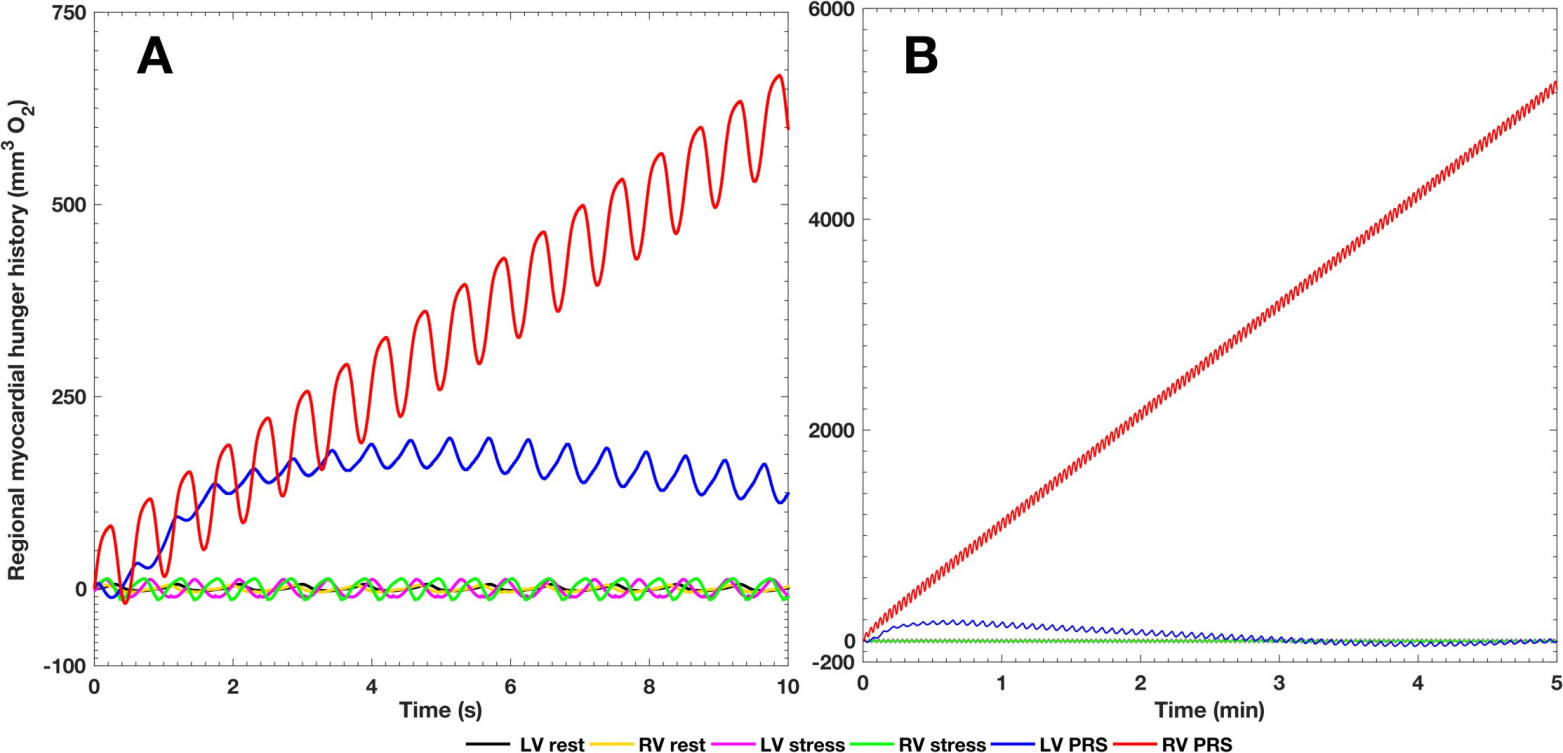
Myocardial oxygen demand/supply mismatch (myocardial hunger) for all three conditions. **Panel A:** Ten seconds of high-resolution results obtained with the multi-scale 3D-0D model. **Panel B:** A longer simulation time (five minutes) obtained with a reduced-order (0D) model reveals an ever increasing RV myocardial hunger and normalization of LV hunger in the long term.

At Rest, coronary resistance was higher in the LCA than in the RCA by just 2%. During Stress, the 39% and 44% drop in LCA and RCA resistance, respectively, was sufficient to match the MVO_2_ demands as shown in Fig 6. However, despite a dramatic vasodilatory response in the RCA during PRS condition, with a 76% reduction in resistance, the CMCM predicted insufficient myocardium oxygen delivery (i.e. myocardial hunger). In turn, the LCA autoregulation predicted a 64% fall in resistance, which was sufficient to counter the supply/demand mismatch after a brief period of ischemia.

## Discussion

### The value of image-based modeling

In-vivo assessment of coronary autoregulation is complex, requiring invasive methodologies that are generally unsuitable for use in patients. Furthermore, the interplay of coordinated control mechanisms present in the coronary microvasculature make computational modeling an appealing prospect for assessing transient events following liver transplantation such as PRS, a serious intraoperative hemodynamic complication associated with significant morbidity and mortality [10]. DSMRI is performed in ALGS patients to assess cardiac risk before liver transplantation. However, while the test might reproduce increases in heart rate and myocardial metabolic demand, it fails to account for the systemic vasodilation and associated coronary perfusion pressure drop during transplantation [26]. Expanding upon previous work [18,29,30], in this study, we used computational modeling to investigate coronary hemodynamics and MVO_2_ during PRS, in combination with image-based and catheterization data under Rest and Stress conditions in an ALGS subject.

### Study findings

There is a scarcity of data on coronary vasodilatory response to pharmacological stress in children, and to our knowledge, no data available in ALGS children. In a small study using positron emission tomography and adenosine stress perfusion, Muzik et al. [31] reported that CFR is impaired in children post-Kawasaki disease compared to healthy volunteers (3.2±0.7 vs 4.6±0.9, respectively). Our computational results showed a much smaller hyperemic CBF response to pharmacological stress (CFR ~ 1.4), notwithstanding an adequate increase in cardiac output elicited by DSMRI. In our study, hyperemic CBF was measured using a different pharmacological agent. Dobutamine, a sympathomimetic amine acting on α/β-adrenoceptors, induces both a positive chronotropic/inotropic response and increases CBF through a metabolic-mediated vasodilation. Whilst the use of an A_2_A receptor agonist such as adenosine is the gold standard for inducing hyperemia, dobutamine assesses the cardiac output response, enabling a more physiological approach for studying demand ischemia. Moreover, adenosine can have vasodilatory effects in the pulmonary vascular bed, affecting RV afterload and consequently RVMVO_2_ and CBF [32].

LVMVO_2_ was slightly higher (6%) than RVMVO_2_ at Rest (Table 2). This pattern was reversed during Stress (LVMVO_2_ was 12% lower than RVMVO_2_) observed after prescribing a 31% increase in heart rate. These observations reflect high RV afterload and are highly pathological. Nevertheless, the CMCM indicated lack of myocardial hunger (Fig 6) through a significant reduction in RCA resistance. During Stress, the RCA flow profile became even more diastolically dominant, reflecting increased systolic compression of the microvasculature. Due to high RV afterload (1.14:1 systolic RV/ aortic pressure ratio) and compensatory hypertrophy (0.91:1 LV-to-RV normalized mass ratio), the RCA Stress diastolic/systolic CBF ratio (2.5:1) was higher than that of the LCA (2.7:1). Additionally, the vasodilatory response during Stress was more prominent in the RCA (44%) than in the LCA (39%). These microcirculatory events suggest that the calibrated CMCM LPN could describe the capillary density inadequacy in patients with RV hypertrophy [33].

We adopted the concept of myocardial hunger [18] to simulate the myocardial demand/supply perfusion mismatch (i.e. ischemia). By assuming a fixed 100% oxygen extraction, the metabolic demand during Stress and PRS was entirely dependent on the CMCM adjustments of microvascular resistances, thus favoring sensitivity over specificity for detecting RV hunger. Of course, there is a minimum physiologically achievable microvascular tone. Based on limited available data [31], we set achievable values for microvasculature resistance (see Table F of the Technical Note in S1 File) that are likely below physiological minima. This means that if we see hunger in the model, we would expect that the myocardial oxygen supply would be insufficient clinically. However, the converse is not necessarily true. In our simulations, the RCA territory vasodilatory response in Stress was sufficient to avoid myocardial hunger. However, in the PRS condition, in the face of the significant drop in MAP (31%), the reduced coronary driving pressure and exhausted vasodilatory reserve (76% resistance decrease) resulted in inadequate post-transplant myocardial perfusion, not anticipated by the DSMRI. Our work suggests that due to abnormal ventriculo-arterial-coronary coupling, RCA flow impairment in this ALGS patient, could limit the RV’s ability to adapt to the hemodynamic changes during liver reperfusion. To the best of our knowledge, there is no specific research addressing this issue in ALGS children.

### Clinical significance

Although several modeling assumptions and simplifications were needed, this study provides a detailed snapshot how CFR impairment in ALGS patients may restrict their ability to adapt to dramatic peri-operative loading changes post-liver transplant. This impaired vasodilatory reserve may be an unrecognized factor determining the immediate and long-term post-transplant outcomes. Our model also emulates coupled ventricular-vascular maladaptations occurring in ALGS that not only increase RVMVO_2_ but also impose an additional burden on the coronary microcirculation autoregulation. We hypothesize that by detecting CFR impairment we could identify a subgroup of patients that despite adequate cardiac output increase with dobutamine stress are at higher risk of cardiac complications post liver reperfusion and could benefit from meticulous optimization of the pre-transplant care (e.g. early diagnosis and endovascular treatment of PPS before RV remodeling; periodic assessment of RV function and size with a MRI; limiting ischaemia–reperfusion injury during organ harvesting) and post-transplant care (e.g. strict hemodynamic monitoring, tailored management of pharmacologic interventions such as vasoactive drugs administration and volume therapy to avoid hypovolemia, as well as excessive cardiac filling resulting in pulmonary edema and deterioration of gas exchange).

Despite the wealth of evidence from experimental animal studies, there is limited patient data available on coronary microvascular pathophysiology and abnormal ventricular-arterial-coronary coupling. Furthermore, because of technical challenges and limitations of in-vivo human studies, the role of reserve exhaustion in the coronary microvasculature in the evolution of diseases that course with chronic increased RV afterload and eventually progress to RV failure is unclear [34]. In patients with pulmonary arterial hypertension, a far common disease, research suggests that increased RV pressure afterload impairs RCA flow and thus myocardial perfusion [22,35], increasing the risk of RV failure [24]. The ultimate validation of our work would require acquisition of CBF data in ALGS children undergoing LT, which is ethically and clinically unfeasible. However, the use of a patient-specific framework of coronary microcirculation such as the one presented, adequately parameterized at baseline and during dobutamine stress to replicate the patient’s hemodynamics, has enabled us to simulate the possible pathophysiological events in the coronary microcirculation during LT, for which it is nearly impossible to obtain such data. These results may then help us to pre-operatively identify ALGS subjects who are at risk of developing RV failure.

### Study limitations

Several limitations need to be acknowledged. The expertise required and the computational cost of the proposed image-based computational model hinders its widespread clinical adoption. Although the results of this paper have been calibrated for the Rest and Stress conditions, the lack of data for the PRS condition makes validation difficult. Therefore, we had to rely on arbitrary estimates of the physiologic limit of the coronary microcirculation vasodilatory response and maximal oxygen extraction. The fluid-structure interaction model utilized in this work relies on a small deformation assumption. While this simplification reduces the computational cost, the deformations of the pulmonary tree and the aorta are such that alternative formulations for large structural displacements might be needed. Interventricular dependence has been noted to be important in pulmonary hypertension patients, with increased RV afterload potentially limiting LV filling. Our heart model did not include this dependence and thus there was no pressure feedback between ventricles. However, we assumed that the results of the virtual PRS Condition are realistic because the computational model was fine-tuned to replicate the patient’s clinical data at Rest and Stress Conditions, for which we had detailed data. This interdependence element could be included in future designs of the LPN heart model in a similar fashion to that described by Arthurs CJ et al. for the CMCM. There, the changes in cardiac workload and metabolic demands estimated from the pressure-volume loops were “broadcast” to the LPN coronary resistors so that a mathematical model therein could attempt to enforce matching of oxygen supply and demand. A potential model that could be used here would be to impose a threshold in the right intraventricular pressure above which this would modify the left heart elastance function to cause LV underfilling. This would likely further reduce the coronary perfusion during PRS.

Finally, the ultimate goal of this manuscript was to demonstrate the application of an image-based methodology to an ALGS patient in order to study potential pathophysiologic responses following PRS, a problem for which it is difficult to acquire in-vivo data. As such, this manuscript is focused on the methodology and the hypothesis generation, and not on obtaining statistical metrics on a larger patient cohort.

## Conclusion

To the best of our knowledge this is the first computational effort to examine altered hemodynamics in ALGS patients. Our model revealed how impaired CFR may restrict adaptive responses in face of reduced aortic perfusion pressure such as that occurring immediately post-transplant. Impaired CFR is likely to result in significant RV myocardial oxygen supply/demand imbalance and could be related to worsened ALGS survival post-transplant. This novel patient-specific experimental computational model can be used to gain insight into the impaired vasoregulatory mechanisms in ALGS, as well as other pulmonary vasculopathies, and aid pre-hepatic transplant cardiac risk stratification. In the future, the predictions of this model could be tested by studying the correlation between outcomes in ALGS patients following liver transplant and MRI quantifications of RCA and LCA flows.

## Supporting information

S1 File Technical note. Description the MRI imaging parameters used, the mathematical model design, the parameterization methods and parameter values, as well as the modeling assumptions made during the study.
(DOCX)

S2 File Movie. Volume-rendered, time and spatially varying vascular hemodynamics of the three simulated conditions.
(MP4)

S3 File Figure. 3D geometry of the aorta and main branches, including the coronaries, and the central pulmonary arteries, segmented from the 3D-SSFP MRI sequence.
(TIF)

S4 File Figure. Instantaneous 3D maps of pressure, velocity and wall shear stress (WSS) at peak systole and peak diastole.
(TIF)

## Abbreviations

ALGS: Alagille’s syndrome
DSMRI: Dobutamine stress MRI
CBF: Coronary blood flow
CFR: Coronary flow reserve
CMCM: Coronary microvasculature control model
LCA: Left coronary artery
LCC: Left coronary circulation
LT: Liver transplantation
LV: Left ventricle
MAP: Mean aortic pressure
MVO_2_: myocardial oxygen consumption
MRI: Magnetic resonance imaging
RCA: Right coronary artery
RHF: Right heart failure
RV: Right ventricle
PPS: Peripheral pulmonary stenosis
SSFP: Steady-state free precession
