# Supplementary material for: Patient-specific modeling of right coronary circulation vulnerability post-liver transplant in Alagille’s syndrome
Source: PLoS One. 2018 Nov 8;13(11):e0205829. doi: 10.1371/journal.pone.0205829 (PMC6224049; doi:10.1371/journal.pone.0205829)
Supplement: S1 File — Description the MRI imaging parameters used, the mathematical model design, the parameterization methods and parameter values, as well as the modeling assumptions made during the study. (DOCX) [file pone.0205829.s001.docx]

**Technical note**

This document describes the MRI imaging parameters used, the mathematical model design, the parameterization methods and parameter values, and the modeling assumptions made during the study.

**MRI Parameters**

The MRI parameters used to acquire the data used are given in Table A.

**Table A. MRI acquisition parameters.**

|  | **Short-axis cine** | **Flows** | **3D morphology** |
| --- | --- | --- | --- |
| **Sequence type** | 2D SSFP | 2D PC | 3D SSFP  (dual-phase) |
| **Number of phases** | 30 | 30 | - |
| **Slice Thickness (mm)** | 7 | 6 | 1.8 |
| **TR (ms)** | 2.7 | 4.3 | 4.5 |
| **TE (ms)** | 1.4 | 2.7 | 2.3 |
| **In-plane pixel size (mm)** | 1.7x1.6 | 2.0x1.6 | 1.3x1.3 |
| **Flip angle** | 60 | 15 | 90 |
| **Signal average** | 1 | 3 | 1 |
| **Respiratory Motion Compensation** | None (breath-hold) | Respiratory gating | iNAV |

**Coupled multidomain model**

The coupled Multidomain model includes 3D models of the systemic and pulmonary arteries, and lumped parameter network (LPN) models representing the left and right hearts, venous systems, systemic and pulmonary vascular beds and coronary microvascular domains. This structure is shown in Fig 1 of the main manuscript. In this supplement, we describe the model design in detail, together with the parameterization procedure. The overview of the parameterization strategy is as follows. First, a family of simplifications of the complete model was determined, to allow model parameters to be identified in the simplest setting, without confounding complexity. Within more complex models, fine-tuning of the LPN parameters was performed to perfect the agreement between the simulation and the patient data. The hierarchy of simplifications was as follows, from most simplified to most complete.

1. Pure-0D open-loop model in which the 3D domains were entirely replaced by a simple resistor-capacitor network – the 3D domain replacement (3DDR). Only the Windkessel, coronary and heart boundary LPNs are included; i.e. the circulatory loop was open. Parameters adjusted using this simplified model were: Windkessel resistance and compliance, ventriculo-arterial valve resistance and inertance (see Fig 1 main manuscript).
2. Pure-0D closed-loop network, which adds the downstream LPNs to the model above (i.e. pulmonary and systemic vascular beds; see Fig 1 of the main manuscript). Parameters adjusted were: venous resistance and compliance, atrio-ventricular valve resistance and inertance, and atrial volume and compliance.
3. Final 3D-0D deformable model, in which the 3DDR model was replaced with deformable 3D image-based models of the pulmonary and aortic domains. Parameters adjusted were: Windkessel compliance and resistance.

**LPN heart model**

We used a 0D LPN model to represent the left and right atrial and ventricular chambers and the delimiting valves (Fig 1 of the main manuscript). A time-varying ventricular elastance function (*E_Ve_*), defined as the ratio of the patient-specific ventricular pressure (*P_Ve_*) and volume (*V_Ve_*) was obtained for the Rest and Stress conditions according to:

$$E_{Ve}\left( t \right)=\frac{P_{Ve}(t)}{V_{Ve}(t)}$$

*V_Ve_ (t)* was obtained by manually contouring the endocardial borders of all cardiac phases of the short-axis cine slices using the cvi^42®^ software (Circle Cardiovascular Inc. Calgary Canada version 5.2.2) with delineation of the papillary muscles (MSV, 5 years of cardiac MRI experience, SCMR level 3 certification). $P_{Ve}(t)$ was available from ventricular pressure catheterisation data. Given that MRI pulses are triggered by the occurrence of an R-wave in the console vectorcardiogram, *V_Ve_ (t)* and *P_Ve_ (t)* were temporally aligned by matching the initial point of the volumetric curve with the corresponding R-wave in the invasive pressure recordings.

The atrio-ventricular and the aortic valves were competent, and so modeled via diodes permitting only forward flow as described before [1]. The pulmonary valve was incompetent, therefore it was modeled as a two-state resistor, having a low resistance to forward flow, and a high but finite resistance to backward flow. The pulmonary valve resistance when the valve is closed, *PV_R,C_* was determined from the patient data by

${PV}_{R, C}=\frac{P_{PA, C}-P_{RV, C}}{Q_{PV, C}}$,

where *P_PA,C_* and *P_RV,C_* are the mean pressures in the pulmonary artery and the right ventricle respectively, during the period when the valve is closed; *Q_PV_*_,C_ is the mean flow through the pulmonary valve during the same period. The resistance of the valve in the open state, ${PV}_{R, O}$, was set to 3.00 x 10^-3^ g/(mm⁴·s).

Valvular inertance (L) was calculated according to the equation [2]:

$$L=\frac{\rho l}{A}$$

where *ρ* is blood density; and *l* and A the length and area of the valve respectively. These were measured from cine images and 2D through-plane flow data at the valve level. Values are presented in Table B.

**Table B.** **Parameters for all heart valves. VAV, ventriculo-arterial valves. AVV, atrioventricular valves.**

|  | VAV inertance  g^.^mm^-4^ | AVV inertance  g^.^mm^-4^ | VAV resistance g/(mm⁴·s) | | AVV resistance  g/(mm⁴·s) | |
| --- | --- | --- | --- | --- | --- | --- |
|  |  |  | Open | Closed | Open | Closed |
| **LV** | 2.61 x 10^-5^ | 3.69 x 10^-5^ | 2.00 x 10^-3^ | ∞ | 1.00 x 10^-3^ | ∞ |
| **RV** | 6.90 x 10^-6^ | 8.90 x 10^-6^ | 3.00 x10^-3^ | 1.80 x 10^-2^ | 6.00 x10^-3^ | ∞ |

Both the left and right atria were modeled as passive compartments with a constant elastance (*E_LA_* and *E_RA_*) calculated using the available invasive pressure data and atrial volumes (biplane method) as detailed in Table C.

**Table C.** **Elastance parameters and approximate volumes for the atria*.***

|  | **Elastance**  Pa/mm^3^ | **Volume**  mm^3^ |
| --- | --- | --- |
| **Right atrium** | 3.52 x 10^-2^ | 4.00 x 10^4^ |
| **Left atrium** | 1.40 x 10^-1^ | 2.25 x 10^4^ |

**LPN model of the peripheral systemic and pulmonary vasculature**

At each outlet of the 3D domain of the aorta and pulmonary arteries a 3-element Windkessel was coupled to represent the downstream vasculature (Fig 1 of the main manuscript). The parameters for the proximal and distal resistor and compliance at outlet *i* (*R_p,i_* and *R_d,i_*, and $C_{i}$ respectively) were computed using the available patient data and the 1D non-linear theory considerations described by Xiao et al [3], and assuming minimization of wave-reflections at the outlet faces.

For the systemic and pulmonary circulations, the respective total peripheral resistance $R_{T, S}$ and $R_{T, P}$ were computed using$R_{T, j}= \frac{P_{mean,j}}{Q_{mean}}$, where $P_{mean,j}$ is the mean pressure in the aortic (*j=S*) or pulmonary (*j=P*) root, and $Q_{mean}$ the mean aortic or pulmonary flow as derived from the 2D through-plane phase contrast MRI (2D PC-MRI). We then apportion the total resistance to the individual outlets using

$R_{i}=R_{T, j}\frac{Q_{mean}}{Q_{i}}$,

where *j* is selected according to whether the *i-th* outlet is on the pulmonary or systemic side.

The proximal resistance *R_p,i_*, the distal resistance *R_d,i,_* and the diastolic wave speed $C_{diast}^{i}$ at outlet *i*, are determined using

$C_{diast}^{i}= \frac{a_{2}}{\left( 2R_{diast}^{i} \right)^{b_{2}}}$,

$R_{p,i}=\frac{\rho C_{diast}^{i}}{A_{diast}^{i}}$,

$R_{i}=R_{p,i}+R_{d,i}$,

where $A_{diast}^{i}$ and $R_{diast}^{i}$are the diastolic area and radius of outlet *i* respectively*,* measured using the diastolic phase of the 3D-SSFP MRI. The remaining terms are constants: *a_2_*=13.3, *b_2_*_=_0.3, and the blood density *ρ=*0.00106 g·mm^-3^.

Windkessel compliances were calculated by writing

$C_{T,j}=C_{C,j}+C_{P,j}$,

with *C_T, j_* the total compliance of the systemic (*j=S*) or pulmonary (*j=P*) circulation, and *C_P, j_* and $C_{C,j}$ the total peripheral (LPN) and 3D domain compliances on each side, respectively. *C_T, j_* is computed from data using

$C_{T,j}=\frac{Q_{max,j}-Q_{min,j}}{P_{syst,j}-P_{diast,j}}{\Delta t}_{Qmin, j}^{Qmax}$,

where *Q_max, j_* and *Q_min, j_* are the maximum and minimal flow into domain *j,* measured from the 2D PC-MRI, ${\Delta t}_{Qmin, j}^{Qmax}$ the time interval between *Q_max, j_* and *Q_min, j_*, and *P_syst ,j_* and *P_diast,j_* the systolic and diastolic root pressures on side *j*, respectively. $C_{C,j}$ was computed as follows. Let $L^{i}$ be the segment length calculated from the 3D-SSFP anatomical data, from the point where it branches from its parent vessel to the outlet (for the aorta, from the root to the aortic outlet at the diaphragmatic level). Then the compliance of vessel branch *i* of the 3D domain is given by

$C_{c}^{i}=\frac{A_{diast}^{i}L^{i}}{\rho\left( C_{diast}^{i} \right)^{2}}$*.*

$C_{C,j}$ is then the sum of $C_{c}^{i}$ over all branches *i* belonging to circulation *j* (pulmonary or systemic)*.*

The peripheral compliance at outlet *i* $C_{P}^{i}$ was computed as

$C_{P}^{i}=C_{P,j}\frac{Q_{i}}{Q_{mean}}\left( \frac{R_{p, i}+R_{d,i}}{R_{d,i}} \right)$*,*

where outlet *i* is part of circulation *j, Q_mean_* is the mean cardiac output per unit time, and $Q_{i}$ is the mean expected flow through outlet *i* per unit time.

To close the circulatory loop, the distal resistor of each 3-element Windkessel model was coupled to a downstream LPN circuit representing the arterioles, venules and veins, with the final venous compartment connected to the right and left atrium respectively (see Fig 1 of the main manuscript). The initial parameters for this circuit were derived from previous work from Lau et al [1] and iteratively adjusted in order to match the available patient hemodynamic data (Tables D and E):

**Table D. Windkessel parameters at all outlets. R, right pulmonary branches. L, left pulmonary branches.**

|  | **Windkessel (W)** | | **Proximal resistor (Rp)**  g/(mm⁴·s) | **Capacitor (C)**  mm⁴·s²/g | **Distal resistor (Rd)** g/(mm⁴·s) |
| --- | --- | --- | --- | --- | --- |
| **Rest** | **Systemic** | **BCT** | 4.00 x 10^-4^ | 1.29 | 4.31 x 10^-2^ |
|  |  | **LCC** | 9.80 x 10^-4^ | 6.17 x 10^-1^ | 9.06 x 10^-2^ |
|  |  | **LSA** | 9.90 x 10^-4^ | 6.14 x 10^-1^ | 9.09 x 10^-2^ |
|  |  | **Aorta^1^** | 2.50 x 10^-4^ | 1.78 | 2.88 x 10^-2^ |
|  | **Pulmonary** | **R1** | 1.64 x 10^-1^ | 2.34 x 10^-1^ | 2.08 x 10^-1^ |
|  |  | **R2** | 1.94 x 10^-1^ | 2.00 x 10^-1^ | 2.47 x 10^-1^ |
|  |  | **R3** | 3.77 x 10^-1^ | 1.03 x 10^-1^ | 4.80 x 10^-1^ |
|  |  | **R4** | 1.59 x 10^-1^ | 2.44 x 10^-1^ | 2.02 x 10^-1^ |
|  |  | **R5** | 1.97 x 10^-1^ | 1.97 x 10^-1^ | 2.50 x 10^-1^ |
|  |  | **R6** | 2.47 x 10^-1^ | 1.57 x 10^-1^ | 3.14 x 10^-1^ |
|  |  | **R7** | 2.72 x 10^-1^ | 1.42 x 10^-1^ | 3.46 x 10^-1^ |
|  |  | **R8** | 1.58 x 10^-1^ | 2.45 x 10^-1^ | 2.01 x 10^-1^ |
|  |  | **L1** | 2.72 x 10^-1^ | 1.42 x 10^-1^ | 3.46 x 10^-1^ |
|  |  | **L2** | 2.54 x 10^-1^ | 1.52 x 10^-1^ | 3.23 x 10^-1^ |
|  |  | **L3** | 4.82 x 10^-1^ | 8.00 x 10^-2^ | 6.13 x 10^-1^ |
|  |  | **L4** | 2.80 x 10^-1^ | 1.38 x 10^-1^ | 3.57 x 10^-1^ |
|  |  | **L5** | 4.82 x 10^-1^ | 8.00 x 10^-2^ | 6.14 x 10^-1^ |
|  |  | **L6** | 4.20 x 10^-1^ | 9.20 x 10^-2^ | 5.35 x 10^-1^ |
| **Stress** | **Systemic** | **BCT** | 6.15 x 10^-7^ | 2.13 x 10^-1^ | 5.18 x 10^-4^ |
|  |  | **LCC** | 1.01 x 10^-6^ | 1.55 x 10^-1^ | 7.75 x 10^-4^ |
|  |  | **LSA** | 2.46 x 10^-6^ | 7.40 x 10^-2^ | 1.63 x 10^-3^ |
|  |  | **Aorta^1^** | 2.48 x 10^-6^ | 7.37 x 10^-2^ | 1.64 x 10^-3^ |
|  | **Pulmonary** | **R1** | 2.46 x 10^-1^ | 8.19 x 10^-3^ | 3.43 x 10^-1^ |
|  |  | **R2** | 2.91 x 10^-1^ | 7.00 x 10^-3^ | 4.08 x 10^-1^ |
|  |  | **R3** | 5.66 x 10^-1^ | 3.61 x 10^-3^ | 7.92 x 10^-1^ |
|  |  | **R4** | 2.39 x 10^-1^ | 8.54 x 10^-3^ | 3.33 x 10^-1^ |
|  |  | **R5** | 2.96 x 10^-1^ | 6.90 x 10^-3^ | 4.13 x 10^-1^ |
|  |  | **R6** | 3.71 x 10^-1^ | 5.50 x 10^-3^ | 5.18 x 10^-1^ |
|  |  | **R7** | 4.08 x 10^-1^ | 4.97 x 10^-3^ | 5.71 x 10^-1^ |
|  |  | **R8** | 2.37 x 10^-1^ | 8.58 x 10^-3^ | 3.32 x 10^-1^ |
|  |  | **L1** | 4.08 x 10^-1^ | 4.97 x 10^-3^ | 5.71 x 10^-1^ |
|  |  | **L2** | 3.81 x 10^-1^ | 5.32 x 10^-3^ | 5.33 x 10^-1^ |
|  |  | **L3** | 7.23 x 10^-1^ | 2.80 x 10^-3^ | 1.01 |
|  |  | **L4** | 4.20 x 10^-1^ | 4.83 x 10^-3^ | 5.89 x 10^-1^ |
|  |  | **L5** | 7.23 x 10^-1^ | 2.80 x 10^-3^ | 1.01 |
|  |  | **L6** | 6.30 x 10^-1^ | 3.22 x 10^-3^ | 8.83 x 10^-1^ |

^1^At diaphragm level. The components referred to are shown in Fig 1 of the main manuscript, and the outlet labels are shown in Fig A of this supplement.

**Fig A. The segmented 3D solid representing the imaged central systemic and pulmonary arteries, with the outlet names specified. Further views of the geometry can be found in S3 File (supporting information).**

| 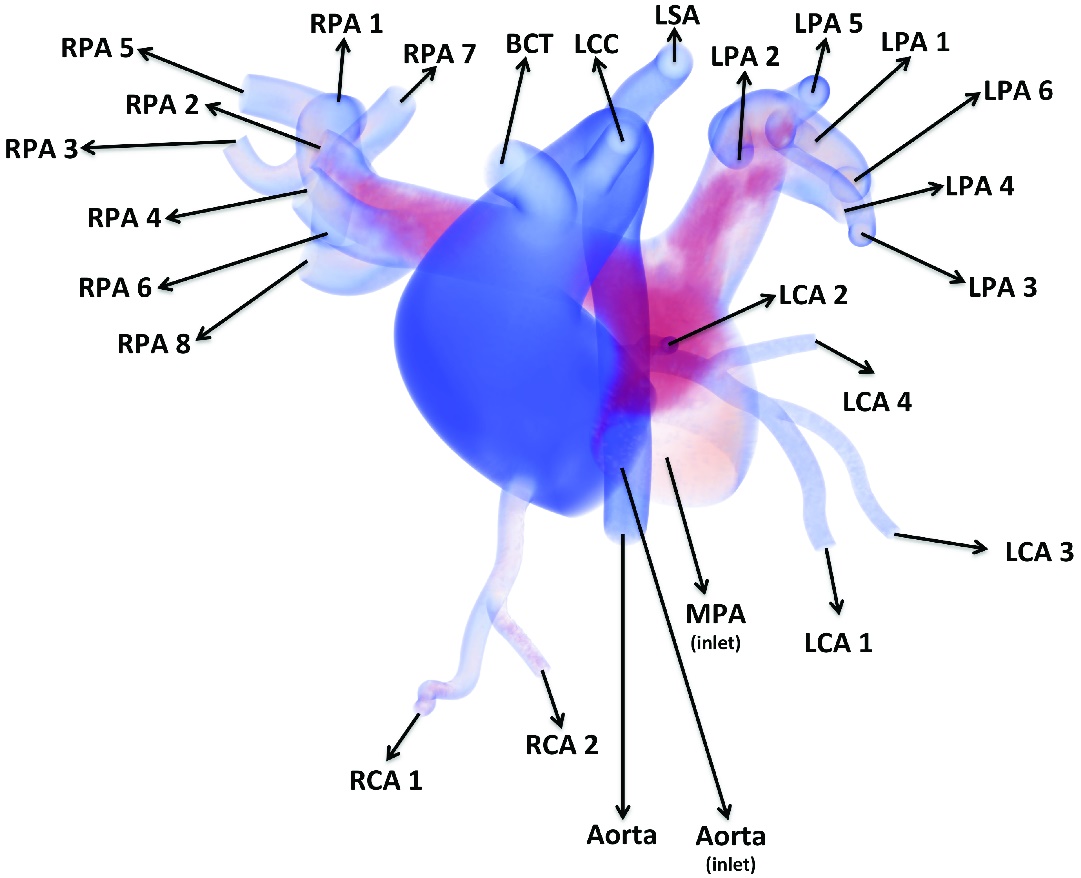 |
| --- |
|  |

**Table E.** **Systemic and pulmonary vascular bed compartment LPN parameters***.*

| **Compartments** | | **Arterioles** | **Venules** | **Veins** |
| --- | --- | --- | --- | --- |
| **Resistance**  g/(mm⁴·s) | **Systemic vascular bed** | 8.89 x 10^-2^ (R1) | 3.56 x 10^-3^ (R2) | 2.97 x 10^-3^ (R3) |
|  | **Pulmonary vascular bed** | 1.40 x 10^-4^ (R1) | 5.54 x 10^-7^ (R2) | 9.9 x 10^-8^ (R3) |
| **Compliance**  mm⁴·s²/g | **Systemic vascular bed** | 8.9 x 10^-3^ (C1) | 3.60 x10^-4^ (C2) | 2.97 x 10^-4^ (C3) |
|  | **Pulmonary vascular bed** | 1.40 x 10^-7^ (C1) | 2.97 x 10-4 (C2) | 9.0 x 10^-8^ (C3) |

The components referred to are shown in Fig 1 of the main manuscript.

**LPN Coronary model with Controlled Microvascular Resistance**

A coronary LPN was attached to each of the coronary outlets of the 3D domain to complete the closed loop. The resistance of the coronary microcirculation was controlled by a coronary microvasculature control model (CMCM), which integrates the aggregate behavior of the neural, local metabolic, myogenic, and wall shear stress control mechanisms acting in the coronary circulation to match the myocardial oxygen supply with the demand, assuming a constant level of coronary oxygen extraction. This model is capable of reproducing invasive patient data during changes in cardiac workload [4]. The ability to predict CBF adaptations in response to changes in the myocardial oxygen demand and the coronary perfusion pressure gradient permits simulation of the coronary response to a generalized vasodilation state and enables specific probing of the complex coronary microcirculatory responses.

The CMCM model is based on a hypothetical concept of myocardial “hunger” (oxygen supply error signal due to the mismatch between myocardial oxygen supply and demand) and a control system that acts to counter any such hunger. It adjusts the resistance of a proximal (R_p) and a distal resistor (R_d) to model feedforward sympathetic adrenergic control of the CBF. The α-vasoconstriction that dominates in vessels of diameters >100μm was represented by model adjustment of R_p, and the feedforward β-vasodilation and feedback metabolic control, predominantly affecting vessels <100 μm, was modeled by adjustment of R_d [5].

Coronary flow results are reported at the stable periodic (patho)physiological states attained after perturbation of the circulatory system (e.g. dobutamine infusion or systemic vasodilation). The cycle-mean values of these parameters, once periodicity was achieved, are presented in Table F.

**Modeling Assumptions**

A number of modeling assumptions were made.

1. Dependent upon the condition being simulated, constant O_2_ extraction per unit of volume of blood delivered in both the left and right coronary circulation was assumed, resulting in the myocardial oxygen delivery being solely dependent on changes in CBF. Under resting physiological conditions, the LV oxygen extraction is nearly at its maximum (~80%), so the LV myocardial oxygen supply increases are predominantly provided by increasing CBF. RV oxygen extraction at rest is lower (~40%), so RV oxygen supply can be increased either by increasing CBF, or by increasing oxygen extraction [4,6]. Therefore, for the Rest condition, we assumed oxygen extraction of 40% for the RCA and 80% for the LCA, whereas for the Stress and PRS conditions, we assumed 100% extraction for both coronary circulations. By design, this approach favors sensitivity over specificity for detecting RV hunger. Physiologically, the relative contributions of RCA CBF and oxygen extraction to balance increased RV oxygen demand in the face of pulmonary hypertension remain uncertain, but it appears the former mechanism is more pronounce [6].
2. The myocardial oxygen demand was computed from the cardiac workload, determined from the 0D heart model. As proposed by Suga H [7], this is a good approximation as oxygen consumption per heartbeat reflects the ventricular work as represented by the ventricular pressure-volume area, integrating wall tension, shortening and contractility [7,8]. This computed oxygen demand was used as input to the CMCM.
3. It was assumed that the RCA supplies the entire RV myocardium, without any contribution to the LV. This RCA perfusion territory might not perfectly represent the reality, but this simplifying assumption is sufficient for our purposes.
4. The extravascular compression experienced by the intramyocardial vessels was modeled by imposing the instantaneous left and right ventricular pressures upon the corresponding coronary LPN. This allows reproduction of the coronary systolic flow impediment in a manner directly dependent upon the ventricular pressure.

**Table F.** **Coronary LPN resistors and capacitors parameters.**

| **Coronary** | | **R_d**  g/(mm⁴·s) | **R_i**  g/(mm⁴·s) | **R_e**  g/(mm⁴·s) | **Ci**  mm⁴·s²/g | **Ce**  mm⁴·s²/g |
| --- | --- | --- | --- | --- | --- | --- |
| **Rest** | **R1** | 8.84 | 1.07 | 1.82 | 3.34 x 10^-3^ | 3.92 x 10^-4^ |
|  | **R2** | 9.75 | 1.18 | 2.01 | 3.08 x 10^-3^ | 3.61 x 10^-4^ |
|  | **L1** | 1.28 x 10^1^ | 1.74 | 2.96 | 2.04 x 10^-3^ | 2.39 x 10^-4^ |
|  | **L2** | 2.56 x 10^1^ | 3.47 | 5.88 | 1.05 x 10^-3^ | 1.23 x 10^-4^ |
|  | **L3** | 2.18 x 10^1^ | 1.75 | 4.32 | 1.43 x 10^-3^ | 1.68 x 10^-4^ |
|  | **L4** | 2.05 x 10^1^ | 2.82 | 4.78 | 1.29 x 10^-3^ | 1.51 x 10^-4^ |
| **Stress** | **R1** | 7.32 | 5.92 x 10^-1^ | 1.82 | 4.38 x 10^-2^ | 5.13 x 10^-3^ |
|  | **R2** | 8.19 | 6.53 x 10^-1^ | 2.01 | 2.23 x 10^-2^ | 2.23 x 10^-2^ |
|  | **L1** | 1.21x 10^1^ | 9.63 x 10^-1^ | 2.96 | 3.03 x 10^-2^ | 3.55 x 10^-3^ |
|  | **L2** | 2.43 x 10^1^ | 1.91 | 5.88 | 2.73 x 10^-2^ | 3.20 x 10^-3^ |
|  | **L3** | 1.76 x 10^1^ | 1.40 | 4.31 | 7.91 x 10^-2^ | 9.28 x 10^-3^ |
|  | **L4** | 1.93 x 10^1^ | 1.55 | 4.78 | 7.21 x 10^-2^ | 8.45 x 10^-3^ |
| **PRS** | **R1** | 1.36 | 5.92 x 10^-1^ | 1.82 | 4.38 x 10^-2^ | 5.13 x 10^-3^ |
|  | **R2** | 1.49 | 6.53 x 10^-1^ | 2.01 | 2.23 x 10^-2^ | 2.23 x 10^-2^ |
|  | **L1** | 3.77 | 9.63 x 10^-1^ | 2.96 | 3.03 x 10^-2^ | 3.55 x 10^-3^ |
|  | **L2** | 7.86 | 1,91 | 5.88 | 2.73 x 10^-2^ | 3.20 x 10^-3^ |
|  | **L3** | 5.55 | 1.40 | 4.31 | 7.91 x 10^-2^ | 9.28 x 10^-3^ |
|  | **L4** | 5.99 | 1.55 | 4.78 | 7.21 x 10^-2^ | 8.45 x 10^-3^ |

R_d, distal intramyocardial resistor. R_i, intramyocardial resistor. R_e, epicardial resistor. Ci, intramyocardial vessel capacitor. Ce, epicardial vessel capacitor. R, right coronary artery branches. L, left coronary artery branches.

**Computational Simulations**

Analyses were run using a time-step size of 0.0005 seconds on 128 cores of a 640 core SGI Altix UV High Performance Computer (HPC) for a total simulation time of 150 hours per case. Convergence to a cycle-to-cycle periodic state was achieved after 100,000 time steps (i.e., 50 seconds of physical time). Manual mesh refinement was performed for each state, and a global combined continuity and momentum residual of 1x10^-3^ was considered. As detailed previously, we used a strategy for gradually increasing the complexity of the final coupled multidomain model starting with a pure-0D open-loop model and then moving to a 0D-3D closed-loop model, iteratively adjusting parameters such as Windkessel resistance and compliance, among others, and finally increasing mesh density to best match simulation and the patient data. Once we found that with subsequent mesh refinements there was no change in the computational results (e.g. differences between iterations were smaller than 5% in mean and pulse pressure in the aorta and pulmonary systems, mean pulmonary and aortic pressure, ventricular volumes and pressure), we assumed that convergence was attained. This entailed using a final mesh with around 5 times as many elements as used initially (from ~ 300,000 elements initially to ~ 1,700,000 elements in the final simulation mesh).

Some initial numerical instabilities, mainly at the 0D-3D heart valve interfaces, required the inclusion of an influx coefficient with a factor beta=0.2. The stabilization was attained as detailed by Bazilevs Y et al. (2009) [9], with the coefficient beta as described in Moghadam ME et al. (2011) [10]. The value of beta was empirically found to provide numerical stability.

**REFERENCES:**

1. Lau KD, Figueroa CA. Simulation of short-term pressure regulation during the tilt test in a coupled 3D–0D closed-loop model of the circulation. *Biomech Model Mechanobiol*. 2015. doi:10.1007/s10237-014-0645-x

2. Broomé M, Maksuti E, Bjällmark A, Frenckner B, Janerot-Sjöberg B. Closed-loop real-time simulation model of hemodynamics and oxygen transport in the cardiovascular system. *Biomed Eng Online*. 2013. doi:10.1186/1475-925X-12-69

3. Xiao N Alberto Figueroa C AJ. A systematic comparison between 1-D and 3-D hemodynamics in compliant arterial models. *Int J Numer Method Biomed Eng 2014 Feb;30(2)204-31*. 2014.

4. Arthurs CJ, Lau KD, Asrress KN, Redwood SR, Figueroa CA. A mathematical model of coronary blood flow control: simulation of patient-specific three-dimensional hemodynamics during exercise. *Am J Physiol Hear Circ Physiol*. 2016;310(9):H1242-58. doi:10.1152/ajpheart.00517.2015

5. Chilian WM, Layne SM, Eastham CL, Marcus ML. Heterogeneous microvascular coronary alpha-adrenergic vasoconstriction. *Circ Res*. 1989;64(2):376-388. https://www.ncbi.nlm.nih.gov/pubmed/2563238.

6. Zong P, Tune JD, Downey HF. Mechanisms of oxygen demand/supply balance in the right ventricle. *Exp Biol Med*. 2005. doi:10.1177/153537020523000801

7. Suga H. Cardiac energetics: from E(max) to pressure-volume area. *Clin Exp Pharmacol Physiol*. 2003;30(8):580-585. https://www.ncbi.nlm.nih.gov/pubmed/12890183.

8. Suga H. Ventricular energetics. *Physiol Rev*. 1990;70(2):247-277. https://www.ncbi.nlm.nih.gov/pubmed/2181496.

9. Bazilevs Y, Gohean JR, Hughes TJR, Moser RD, Zhang Y. Patient-specific isogeometric fluid-structure interaction analysis of thoracic aortic blood flow due to implantation of the Jarvik 2000 left ventricular assist device. *Comput Methods Appl Mech Eng*. 2009. doi:10.1016/j.cma.2009.04.015

10. Esmaily Moghadam M, Bazilevs Y, Hsia TY, Vignon-Clementel IE, Marsden AL. A comparison of outlet boundary treatments for prevention of backflow divergence with relevance to blood flow simulations. In: *Computational Mechanics*. ; 2011. doi:10.1007/s00466-011-0599-0
