# Supplementary figures and images for: Patient-specific modeling of right coronary circulation vulnerability post-liver transplant in Alagille’s syndrome
Source: PLoS One. 2018 Nov 8;13(11):e0205829. doi: 10.1371/journal.pone.0205829 (PMC6224049; doi:10.1371/journal.pone.0205829)

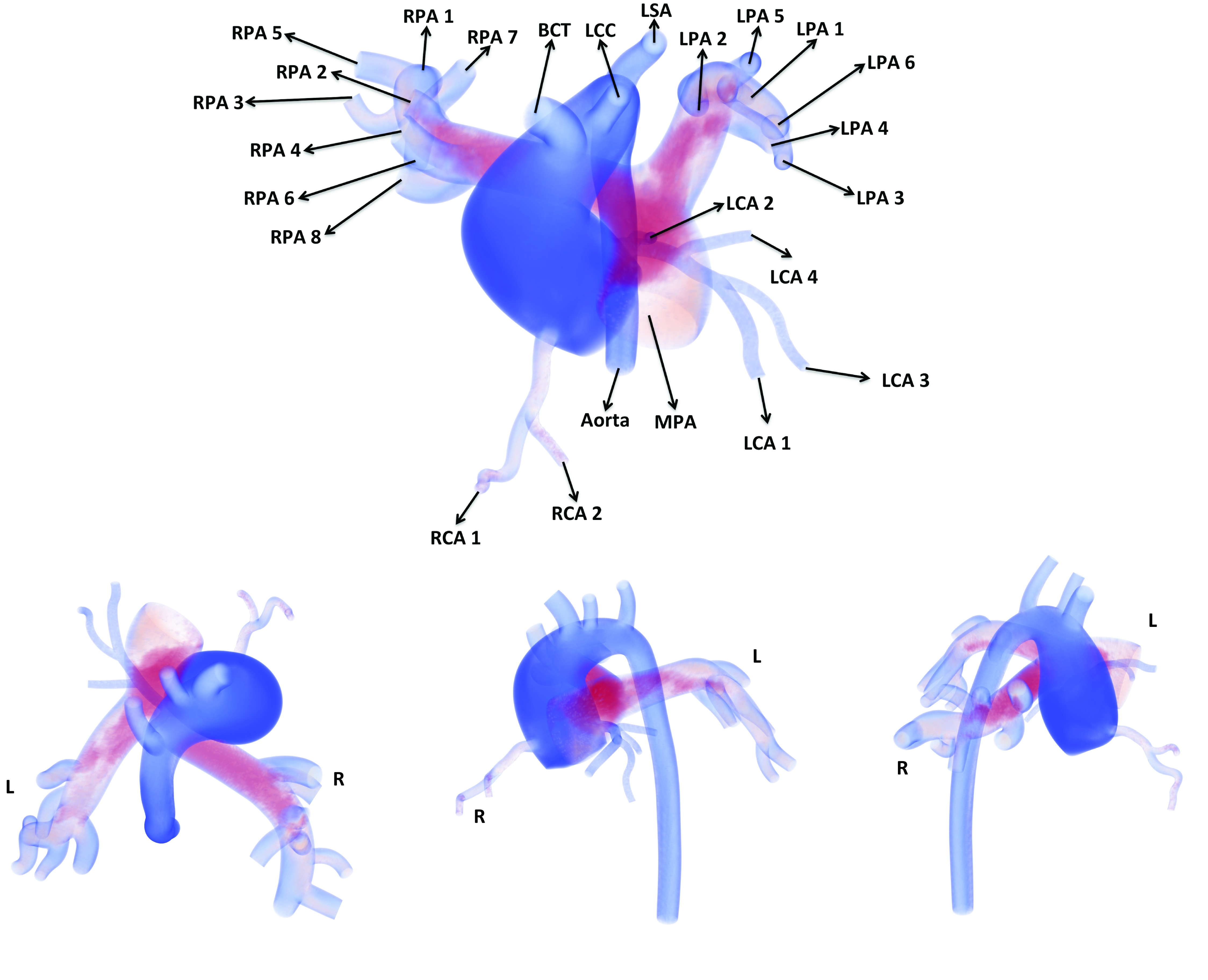

Supplement: S3 File — 3D geometry of the aorta and main branches, including the coronaries, and the central pulmonary arteries, segmented from the 3D-SSFP MRI sequence. (TIF) [file pone.0205829.s003.tif]

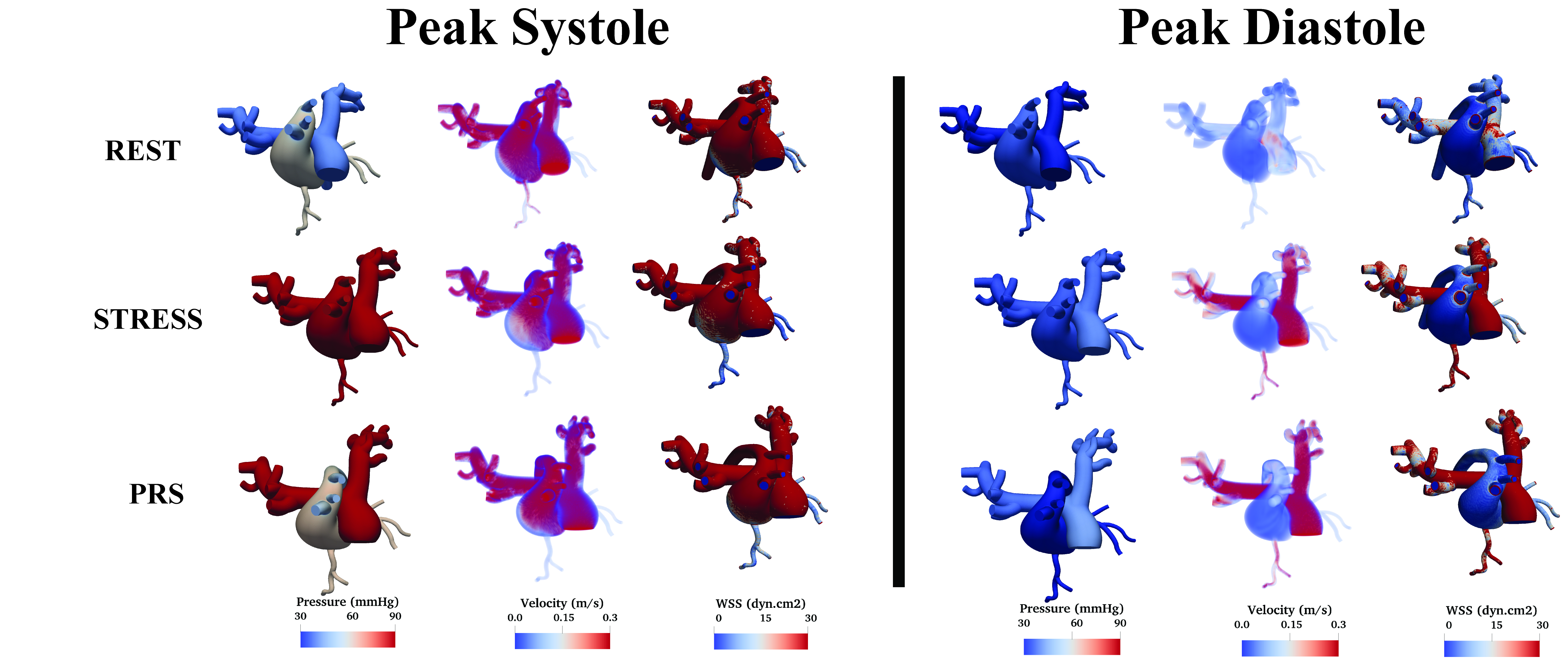

Supplement: S4 File — Instantaneous 3D maps of pressure, velocity and wall shear stress (WSS) at peak systole and peak diastole. (TIF) [file pone.0205829.s004.tif]
